# Functionalization of La_0.33_Ca_0.67_MnO_3_ with biologically active small ligand at room temperature

**DOI:** 10.1016/j.mex.2019.03.004

**Published:** 2019-03-22

**Authors:** A. Edobor-Osoh, P. de la Presa, B.I. Ita, K.O. Ajanaku, F.E. Owolabi, S.J. Olorunshola

**Affiliations:** a Department of Chemistry, Covenant University, Ota, Ogun State, Nigeria; b Department of Pure and Applied Chemistry, Calabar, Cross River State, Nigeria; c Instituto de Magnetismo Applicado, UCM-ADIF-CSIS, Madrid, Spain; d Department of Biological Sciences, Covenant University, Ogun State, Nigeria

**Keywords:** Functionalization of manganite, Functionalization, Manganite, Ethyl 4-nitrobenzoate, Nanoparticles, Antimicrobial agent

## Abstract

We report the structural, morphological and optical activities of a paramagnetic manganite (La_0.33_Ca_0.67_MnO_3_) synthesized at 900 °C. A simple method of formation of complex was employed. A complex was formed between a pre-prepared manganite dissolved in HCl and reacted with an organic ligand (ethyl 4-nitrobenzoate). The optical and antimicrobial properties of a complex were determined. The Ultraviolet-visible and Fourier-transform infra-red spectroscopy were used in monitoring optical activities of the resulting product. It was observed to absorb in the visible region (205 nm and 256 nm). The peaks observed from the infra-red spectra indicated that the reaction occurred at the nitroso end of the ethyl 4-nitrobenzoate. The bacterial inhibitory property of the LCMO-ethyl 4-nitrobenzoate was determined against Pseudomonas aeruginosa, Candida albican, Aspergillus niger and Staphylococcus auerus. It was observed to inhibit the growth of all the microbes with zone of inhibitions of 60 mm, 56 mm, 45 mm and 32 mm, for Pseudomonas aeruginosa, Candida albican, Aspergillus niger and Staphylococcus auerus, respectively.

• The method used was simple and a complex was formed within 6 h without the use of complicated equipment.
• The method requires no heat treatment and can be prepared at room temperature.
• LCMO-ethyl 4-nitrobenzoate was biologically active against *Pseudomonas aeruginosa*, *Candida albican*, *Aspergillus niger* and *Staphylococcus aureus*.

## Graphical abstract

Pictures of the growing crystals.

**Figure:**
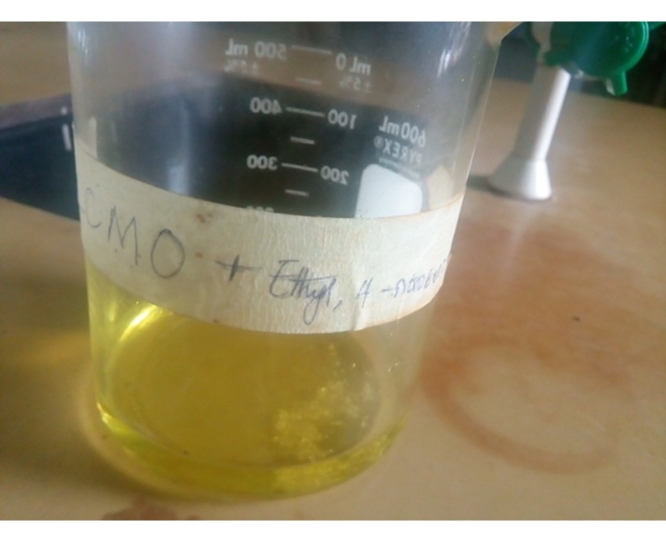

Specifications Table

**Table:** 

**Subject Area:**	Materials Science
**More specific subject area:**	Biomedical nanoparticles
**Method name:**	Functionalization of manganite
**Name and reference of original method:**	1. Giri, A., Makhal, A., Ghosh, B., Ravchaudhuri, A. K. and Pal, S. K. (2014) Functionalization of manganite nanoparticles and their interaction with biologically relevant small ligand: Picosecond time-resolved FRET studies. Nanoscale, 2, 2704-2709 2. Iniama, G., de la Presa, P., Alonso, J. M., Multigner, M., Ita, B. I., Cortés-Gil, R., Ruiz-González, M. L., Hernando, A., and Gonzalez-Calbet, J. M. (2014). Unexpected ferromagnetic ordering enhancement with crystallite size growth observed in La_0.5_Ca_0.5_MnO_3_ nanoparticles. Journal of Applied Physics, 116 (11,390)
**Resource availability:**	NA

## Method details

### Background

Perovskites are compound with a general formula ABO_3_ (A are rare earth metal (lanthanides), B sites have transition metal (Fe, Mn, Cr, etc.); manganites are perovskites with transition element manganese (Mn) as a component of the compound, thereby modifying the general formula to AMnO_3_. Doping of manganites at the A-site with a divalent metal confers interesting intrinsic properties such as magneto-caloric effect, colossal magneto-resistivity, and transport properties [[1], [2], [3], [4], [5], [6], [7], [8], [9]]. These properties have been studied intensely by a number of researchers. Although a lot is known about the structural and morphological properties of the surface of the solid state, however, the nature of the surface of metal oxides in the presence of an aqueous solution has not really understood [10,11]. It is assumed that at pH < 6, the properties of the surface area of the metal does not change, therefore making it possible to functionalize the surface of the metal by modification, whereas at pH > 6 the manganite dissolves into different components; pyrolusite (MnO_2_(s)) and manganese ions Mn^2+^(aq). The complex formed between the dissolved manganite and ethyl 4- nitrobenzoate indicates that the aqueous manganese react readily with the organic ligand. Although a lot is known on the antimicrobial properties of nanoparticle [12]; however, biological activities of manganites generally have been studied by only a few researchers [13,14]. In recent times the stability of dextran coated manganite (La_0.7_Sr_0.3_MnO_3_ (LSMO)) in water and phosphate buffer solution was determined. It was noted that the stability of dextran coated La_0.3_Ca_0.7_MnO_3_ were better than the bare manganite. It was also observed that the specific absorption rate of the dextran coated LSMO was significant and the manganites were not toxic to the L929 cells when introduced [15].

### Reagents used in the study

The reagents used in this study included:

Hydrochloric acid (1 M), concentrated nitric acid, lanthanum oxide, calcium carbonate, manganese carbonate, ethylene glycol, citric acid monohydrate, ethyl 4-nitrobenzoate

#### Equipment used in the study

The equipments used in analyzing the sample are as follows:

Scanning Electron Microscope (JEOL JSM 6400)

X-ray Diffraction (PANalytical X’pert Pro MPD diffractometre)

Magnetic stirrer (Stuart Agar Hot plate/Magnetic stirrer Model Number: AGG3788C)

Autoclave (Thermo-Fischer precision compact oven)

UV-visible Spectrophotometer (Genesys 30 visible spectrophotometre)

Fourier Transform Infra-red Spectrophotometre (Perkin-Elmer λ25 and a Thermo Nicolet FTIR 200 spectrophotometre)

#### Preparation of the samples (Synthesis of the manganite)

1 In preparing the sample, analytical grade La_2_O_3_, MnCO_3_ and CaCO_3_ were stoichiometrically reacted forming a greenish solution, based on method used by [16].
2 La_2_O_3_ was pre-prepared by heating in a furnace for 2 h at 900 °C to remove the water of crystallization embedded in the sample.
3 5 mL of nitric acid was heated up to 50 °C on a hot plate.
4 1.36 g of lanthanum acid was added into the acidic solution on the hot plate until it turned transparent.
5 10 g of citric acid was added to the solution.
6 1.68 g of MnCO_3_ was added to the solution and stirred until the solution became transparent.
7 1.68 g of CaCO_3_ was added into the solution and it was allowed to dissolve completely.
8 10 mL of ethylene was added as a fuel for combustion.
9 The solution was heated until it evaporated to a xerogel.
10 With continuous heating of the xerogel the sample combusted into brown powder.
11 The brown powder was milled for 2 h using agate mortar.
12 The milled sample was heated up for 24 h at 600 °C to remove all organic compounds in the sample.
13 The sample was sintered at 900 °C for 2 h.
14 The structural and morphological properties of the sample were determined using XRD and SEM, respectively.

The proposed equation of reaction based on the precursors used is as described below:

(1) $$\frac{1}{6}La_{2}O_{3}+\frac{2}{3}CaCO_{3}+MnCO_{3}\to La_{0.33}Ca_{0.67}MnO_{3}+CO_{2}↑$$

### Morphological properties of La_0.33_Ca_0.67_MnO_3_

The morphological property of the manganite was determined by using JEOL JSM 6400 Scanning Electron Microscopy (SEM).

1 The samples analyzed by SEM imaging were dry and clean.
2 The oil on the surface of the sample holder was cleaned up before use.
3 A small portion of the sample was taken and gently sprinkled using a small pipette on a carbon tape which was mounted on a sample holder.
4 Blower was used to remove loosely held samples.
5 The samples were coating with a conductive carbon metal (graphene), using a sputter coater to reduce the charging effect before SEM images are taken [17,18].

**Fig. 1:**
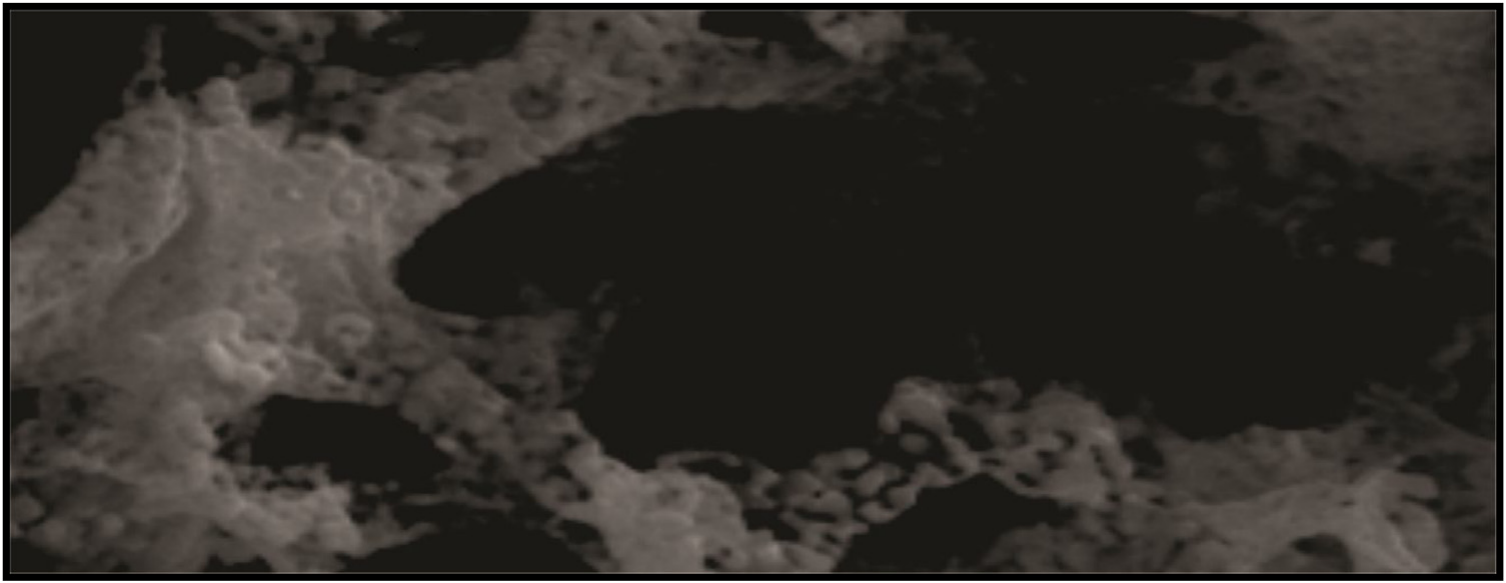
SEM micrograph of LCMO 900.

The surface topography as well as the sizing of the sample was done via SEM. It was determined to be heterogeneously shaped and sized. The sample was observed to be porous as shown in Fig. 1. The nominal composition La_0.32_Ca_0.68_Mn_0.97_O_3_ was determined for the sample. The slight difference between the nominal and experimental composition was probably due unavoidable errors incurred during synthesis.

### Structural properties of the sample

X-ray diffractometre (XRD) of the sample at room temperature was obtained using Cu-K_α_ radiation of wavelength 1.54056 Å in a PANalytical X’pert Pro MPD diffractometre.

1 A minute volume of the sample was tapped onto a glass slide for analysis. The sample was loaded into the XRD instrument and installed by opening the door of the instrument.
2 A quick scan of the structural properties of the sample was done at 10,000–100,000 scan range at 0.030 step size. The mean crystallite size <**D**> was calculated from the Scherer’s formula.
3 Fullprof programme based on Rietveld method [19] was used for phase analysis. This gave the cell dimensions, atomic position, R factor and a model diffraction pattern utilizing PCR file code 56712.

**Fig. 2:**
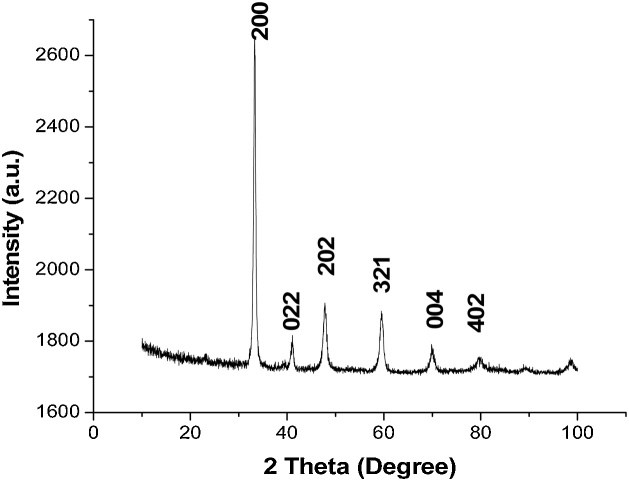
XRD Pattern of La_0.33_Ca_0.67_MnO_3_ oxide annealed at 900 ^o^C.

Fig. 2 shows the XRD diffractograph of La_0.33_Ca_0.67_Mno_3_ broadening of the peaks in Fig. 2 indicates that the sample was nanoscaled. It was also observed that the sample was a single perovskite phase in the orthorhombic, Pnma crystal structure.

The crystallite size was determined by using the Scherer’s Eq. (2):

(2) $$D=\frac{0.9\lambda }{\beta_{FWHM}\text{Cos}\theta }$$

Where, λ =0.154 nm

β_FWHM_ = Full width at half maximum of the X-ray reflection

θ = Position of the highest intensity

#### Surface functionalization of La_0.33_Ca_0.67_MnO_3_ manganite oxide

The samples were synthesized based on a modified version of methods used by [20].

**Fig. 3:**
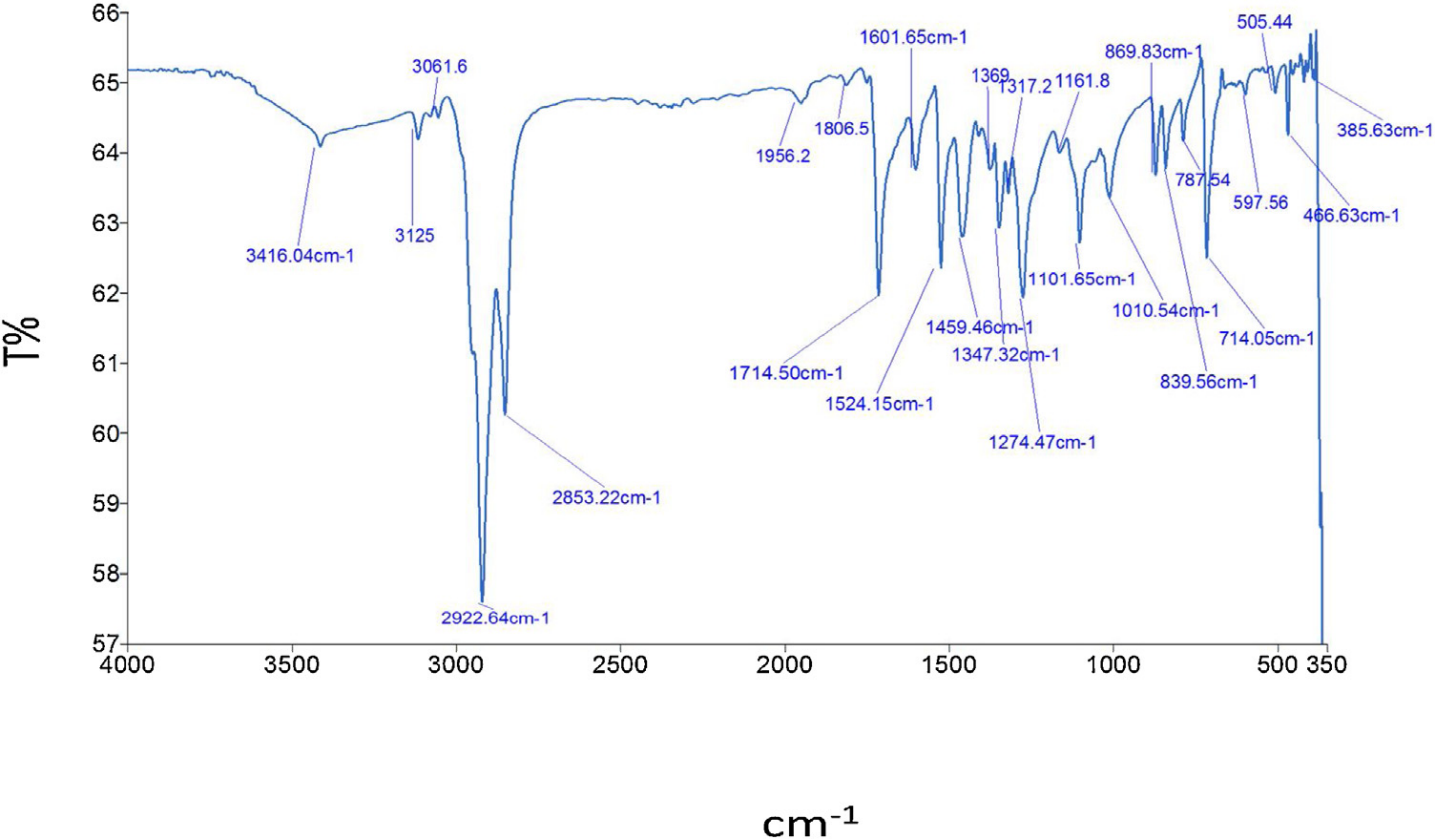
FT-IR image of the LCMO-Ethyl 4-nitrobenzoate complex showing the peak.

1 Pre-prepared La_0.33_Ca_0.67_MnO_3_ was dissolved in 150 mL 1 M HCl with pH 5.6 and stirred homogenously for an hour at room temperature, a greenish yellow solution was observed.
2 The ethyl 4-nitobenzoate was used as purchased from Merck, Germany.
3 The ethyl 4-nitrobenzoate was dissolved in 150 mL of ethanol.
4 150 mL of the ligand solution was divided into five aliquots of 30 mL each.
5 30 mL of the sample was added into 150 mL of the manganite solution while stirring at room temperature for at least 2 h.
6 The synthesis of the complex was monitored using thin layer chromatography (TLC) plates and UV–vis spectrophotometer.
7 After this, the beaker containing the solution was covered with perforated aluminum foil and left undisturbed for two weeks for the crystals to grow.
8 The recovered crystal was filtered and dried.
9 The sample was weighed to obtain the percentage yield.
10 Optical analysis of the sample was done by using Fourier-transform Infra-red and Ultraviolet-visible spectrophotometer. Fig. 3 and Table 1 describe the Fourier Transform infra red peaks. The Ultra-violet peak assignment was done in Table 2. It was utilized to determine the site of reaction between the metal and the ligand.

**Table 1:** The Infra-red peaks observed for the complex formed and the organic ligand (ethyl 4-nitrobenzoate).

Functional Groups	Range (cm^−1^) LCMO-Ethyl 4-nitrobenzoate	Range (cm^−1^) Ethyl 4-nitrobenzoate
C_2_H_5_COO (Ester)	1714.50	1712.00
Ar—C=O	1603.65	1607.00
Ar—N=O	1526.15	1527.00
Mn—O	476.63	–

**Table 2:** Comparison of the ultraviolet-visible wavelength (nm) of ethyl 4-nitrobenzoate and LCMO-ethyl 4-nitrobenzoate.

S/N	Name of Sample	Wavelength (nm) ($\log\varepsilon_{\max}$)	
**1**	Ethyl 4-nitrobenzoate	256 (5.065)	202 (4.512)
**2**	LCMO + Ethyl 4-nitrobenzoate	256 (5.421)	205 (4.972)

#### Antimicrobial analysis of the LCMO-ethyl 4-nitrobenzoate

**Determination of the zone of inhibition**

1 Prior to the analysis the sample was dissolved in a universal solvent (Dimethyl sulphoxide) (DMSO) and the negative control was done.
2 The zone of inhibition (Table 3) and of the dissolved samples was determined by using the well diffusion method as described by [21].

**Table 3:** The zone of inhibition of the La_0.33_Ca_0.67_MnO_3_ –ethyl 4-nitrobenzoate (complex), controls anti-fungal (nitrofuratoin) and anti-bacterial (gentamicin).

Sample	Concentration	*Staphylococcus* *aureus* (ATCC 25923) (mm)	*Aspergillus niger* (cultured) (mm)	*P. aeruginosa* (ATCC 15442) (mm)	*Candida albican* (ATCC 10231) (mm)
La_0.33_Ca_0.67_MnO_3_ – Ethyl 4-nitrobenzoate (Complex)	1000 mg/mL	32	45	60	56
Nitrofuratoin (control)	300 μg/mL	–	32	–	28
Gentamicin (control)	10 μg/disc	25	–	24	–
3 Nutrient agar was used to sub-culture the microorganisms.
4 The agar was heated so as to convert it to liquid state after which it was cooled to 50 °C in a water bath.
5 Streaks of the *Staphylococcus aureus* (ATCC 25923), *Pseudomonas aeruginosa* (ATCC 1544), *Aspergillus niger* (cultured) and *Candida albican* (ATCC 10231) was put into the agar and left alone to set.
6 The petri dish was put in an incubator for 24 h at 37 °C.
7 After this period, holes of about 5 mm to the edge of the plates were drilled into the petri dishes by using a sterile cock borer.
8 200 μl of the dissolved solution was inoculated into the perforated well using a micro pipette.
9 The petri dish was pre-diffused for 30 min and kept in an incubator for 24 h at 37 °C, until an obvious reduction of the potency of the complex was observed.
10 The zone of inhibition of the sample was determined in mm.
11 10 μg/disc of a standard anti-fungal drug (nitrofuratoin) and an antibacterial drug (gentamicin) were used as controls.

**Determination of the minimum inhibition concentration (MIC)**

1 The minimum inhibitory concentration (MIC) of the samples were determined using the broth dilution method as shown in Table 4.

**Table 4:** The minimum inhibitory concentration of the La_0.33_Ca_0.67_MnO_3_ – ethyl 4-nitrobenzoate (complex).

Micro-organism	Minimum inhibitory concentration (mg/mL)
*Candida albican*	31.25
*Staphylococcus aureus*	7.80
*Pseudonomas aeruginosa*	7.81
2 The antibiotic (LCMO-ethyl 4-nitrobenzoate) stock solution was prepared by dissolving in DMSO.
3 Sterile test tubes were used in conducting the test.
4 The antibiotic was diluted in the range of 0–300 mg/mL.
5 A final volume of 1 mL of the dilution was used in the MIC test.
6 Broth suspension of the bacteria was selected from plates that have been incubated for 24 h at 35 °C.
7 1 mL of the diluted antibiotics was added and a positive control tube containing the broth and the microbes was also used
8 The inoculated tubes were incubated for 24 h at 35 °C.
9 The turbidity of the tubes was determined visually.
10 The growth of the microbes in the tubes containing the antimicrobial agent was compared with the amount of growth with no antimicrobials.
11 The lowest concentration that inhibited the growth of the microbes was considered as the minimum inhibitory concentration.

#### Additional information

This method of synthesis was utilized by [20] using a cyclo-mixer for the capped La_0.67_Sr_0.33_MnO_3_-citrate. The capped sample was reacted with 4-nitrophenylanthranilate (NPA). In our study the same method was utilized by using a magnetic stirrer. The optical property of the sample was determined using ultraviolet-visible and Fourier-Transform infra-red (FT-IR) spectrophotometer showed that a complex was formed between the metal and the organic ligand. HCl was used to dissolve the sample at a pH > 6 and the resulting manganese formed a complex with a small organic ligand (ethyl 4-nitrobenzoate). The slight bathochromic shift in the band position 202 nm indicates the coordination reaction, but the optical activity of the product was attributed to the organic ligand [22]. The antimicrobial property of La_0.33_Ca_0.67_MnO_3_-ethyl 4-nitrobenzoate was determined by using *Candida albican*, *Pseudomonas aeruginosa*, *Aspergillus niger* and *Staphylococcus aureus*.
